# Novel *Prosopis juliflora* leaf ethanolic extract as natural antimicrobial agent against food spoiling microorganisms

**DOI:** 10.1038/s41598-021-86509-3

**Published:** 2021-04-12

**Authors:** Iman Saleh, Mohammed H. Abu-Dieyeh

**Affiliations:** Department of Biological and Environmental Sciences, College of Art and Science, Qatar University, P.O. Box 2713, Doha, Qatar

**Keywords:** Antimicrobials, Fungi, Microbiology, Plant sciences, Environmental sciences

## Abstract

Fresh produces spoilage is a worldwide concern that accompany the global increase in food demand. Adverse human health and environmental effects of commercial spoilage control agents are major public concern. In this study, *Prosopis juliflora* leaves and fruit extracts had their antimicrobial activities evaluated against the growth of selected bacteria and yeast, and against mycelial growth and conidial germination of selected mycotoxins-producing fungi. *P. juliflora* water-soluble leaf ethanolic (PJ-WS-LE) extract with its novel extraction method showed the strongest antibacterial activity. Antimicrobial tests showed total inhibition of *Botrytis cinerea*, *Alternaria alternata*, *Bacillus subtilis*, *Staphylococcus aureus* and *Candida albicans* with MICs ranging between 0.125 and 1 mg/ml. Percent inhibition of mycelial growth (PIMG) of the extract was also determined against seven other fungal strains with highest value against *Geotrichum candidum* (66.2%). Even the least affected fungal strain showed alterations in their hyphae and spores exposed to PJ-WS-LE extract when observed using scanning electron microscope (SEM), alterations include exfoliated flakes, pores, vacuolation and applanation. Small-scale fruit bioassays controlled experiment showed high efficacy of the extract in protecting inoculated cherry tomato samples from *B. cinerea* and *A. alternata* infections. In conclusion, PJ-WS-LE extract is a feasible, natural antifungal agent that can replace common anti-spoiling chemicals.

## Introduction

Microbial infections of fruits and vegetables are caused by fungal and bacterial pathogens (spoilage microorganisms) that can be harmful to human and animal health and it can lead to significant financial losses for both manufacturers and consumers^1^. Food sustainability is a major concern worldwide with the growing world population that has led to a tremendous increase in food demand^2^. Chemicals such as antifungal and antimicrobial agents are used for spoilage control of fruits and vegetables, and while these chemical agents help increase the preservation of fresh produce, they also pose a potential threat to human health and cause environmental damage. As a result, various countries around the world are setting a maximum residue limit (MRL) of chemicals on the skin of their exported fruits and vegetables^3^. In addition, spoilage microorganisms are becoming resistant to the commonly used antimicrobial agents^4^. This pushes scientists to further explore natural products to replace chemical applications in the field and at post-harvest levels^5^.

Plant-based raw materials are susceptible to microbial spoilage by fungi and bacteria due to their high nutrient density. Fruits become more prone to fungal contamination during the ripening process where they become rich with carbohydrates and less acidic, creating a highly suitable environment for food spoilage fungi to thrive^6^. Advanced fungal contamination causes visible symptoms on the plants such as detectable change in color and/or texture, hyphal growth and others. Spoiling fungi affect mainly citrus fruits, berries, pome fruits, stone fruits, and tropical and solanaceous fruits^7^. The most common postharvest diseases are caused by fungi species belonging to the following genera: *Penicillium*, *Botrytis*, *Monilinia*, *Rhizopus*, *Alternaria*, *Aspergillus*, *Fusarium*, *Geotrichum*, *Gloeosporium*, and *Mucor*. *Aspergillus, Penicillium, Fusarium* and other fungi are mycotoxin-producing fungi^8^. Infection of fruits and vegetables by mycotoxin-producing fungi poses a major health risk to consumers as cumulative mycotoxins in the body can be mutagenic and carcinogenic and can cause damage to different organs^9^.

Thorough research for spoilage bio-controllers reveals many promising results. Bacteriocins, phytochemicals from plant extracts, natural oils and other biological products have been screened for their antimicrobial properties, yet very few of these tested agents have been patented and made commercially available in the market to replace the chemical agents. Plant extracts are being explored in many studies as a natural bio-controller for food spoilage organisms. Phytochemicals in higher plants are valuable drugs known to cure several diseases^10^. Numerous studies have recently shown the antifungal effect of higher plant extracts which served as fungi-toxic agents against spore germination and/or growth of mycelia^11^. Among the tested plants, *Prosopis* species in general, particularly *P. juliflora*, have been screened for their antioxidant, anti-inflammatory, antibacterial, antifungal, and anti-tumor effects^12,13^. These studies examined the extracts of different parts of the plant while others analyzed purified phytochemicals, yet the studies are not interconnected. Chemical analysis of *P. juliflora* leaves ethanolic extract have shown antioxidant activity with the presence of alkaloids, tannins, flavonoids, coumarins and anthraquinone glycoside^14^. Aqueous extracts of the same leaves have also been evaluated to show similar composition with higher antioxidant activity in some studies^15^.

A successful antifungal product should control pre-existing infections and leave residues that prevent subsequent infections and delay sporulation of the existing spores, thereby reducing economical losses^9,16,17^. In the case of fungi, applying an affordable, natural, and non-hazardous formulation would prevent visible spoilage as well as inhibit the growth of a fungus before the formation of dangerous mycotoxins, providing fresh produce which are safer for consumption.

This research explores the effect of a novel leaf extract of *P. juliflora* as a natural, eco-friendly, and effective antifungal agent to control microbial food spoilage. Coming up with a naturally based anti-spoilage formulation will boost the economy of many countries, enhance food security measures, and solve a worldwide agricultural problem without posing a threat to the environment.

## Results

### Molecular identification of food spoiling fungal isolates

DNA extraction followed by PCR using ITS primers and PCR product sequencing helped in identifying the fungal isolates at the species level. NCBI-BLAST identification results are shown in Table 1.

**Table 1:** PCR products blasting results indicating species of fungal isolates used.

Expected fungal genus (microscopic identification)	Sequence length	Fungal species (molecular identification)	Strain code	Percentage of nucleotides identity (%)
*Aspergillus*	720 bp	*Aspergillus niger*	AnigQU17	97.10
*Penicillium 2*	1115 bp	*Penicillium chrysogenum*	PchrQU17	99.27
*Botrytis*	1006 bp	*Botrytis cinerea*	BcinQU17	98.59
*Fusarium*	904 bp	*Fusarium oxysporum*	FoxyQU17	94.82
*Alternaria*	899 bp	*Alternaria alternata*	AaltQU17	96.38
*Penicillium 1*	1011 bp	*Penicillium citrinum*	PcitQU17	99.02
*Colletotrichum*	677 bp	*Colletotrichum gloeosporioides*	CgloQU17	96.00
*Cladosporium*	1081 bp	*Cladosporium cladosporioides*	CclaQU17	97.44
*Geotrichum*	357 pb	*Geotrichum candidum*	GcanQU17	94.35

### In-vitro antimicrobial effect of PJ-WS-LE extract

#### Antifungal effect

##### Agar diffusion method

**Figure 1:**
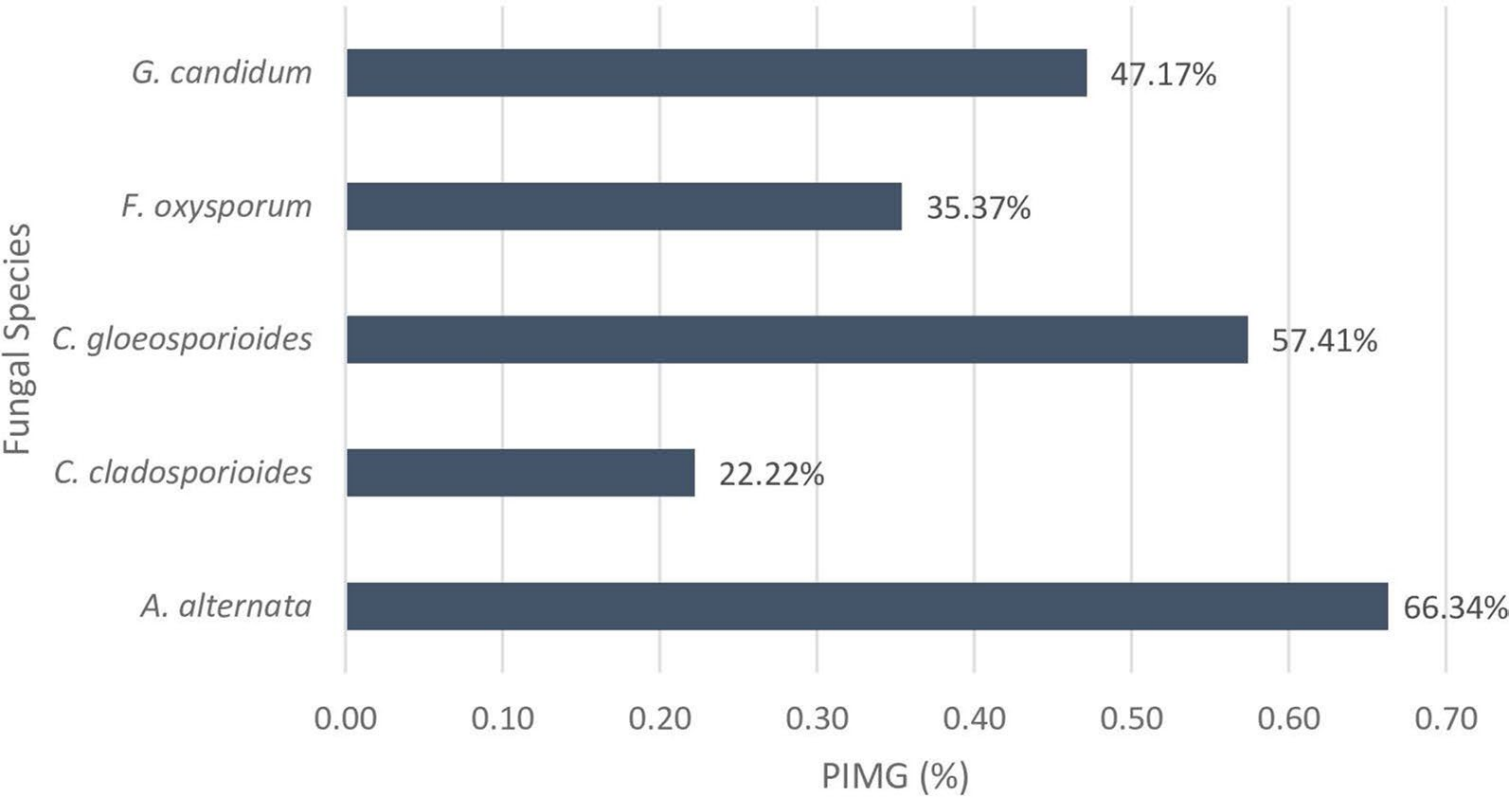
Percent inhibition of mycelial growth (PIMG) of 4% DMSO PDA plates against five fungal strains incubated at 25 °C for 5 days.

**Figure 2:**
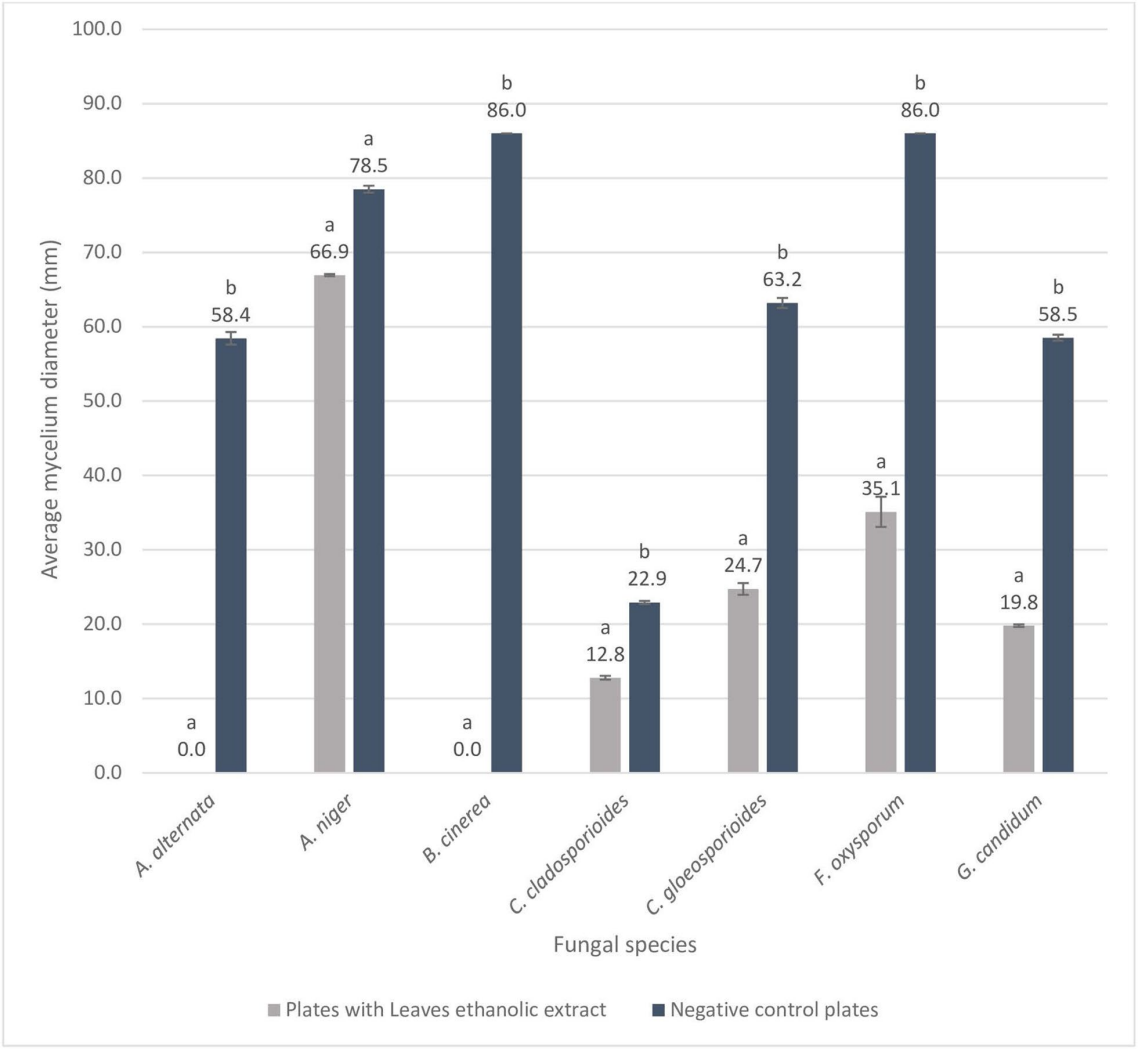
Average mycelium diameter (mm) ± SE (N = 4) of seven fungal species in the presence and absence of PJ-WS-LE extract after 5 days incubation at 25 °C. ^ab^Treatment columns with different letters have their values significantly different as shown by t-test for data of fungal species.

Ethanolic and aqueous extracts of *P. juliflora* leaves and fruits were screened for their antifungal and antibacterial activity. Freshly blended leaves and fruits were also tested for their effectiveness. Preliminary screening showed that ethanolic extract of leaves had the best antimicrobial effect which led to it being further tested throughout this study. Leaves ethanolic extract was dissolved in two solvents (a) 4% Dimethyl sulfoxide (DMSO) and (b) sterile distilled water, both solutions showed high anti-fungal activity. However, negative control batch of PDA plates with 4% DMSO proved the toxic effect of the solvent discussed in the literature by showing high antifungal activity (Fig. 1). Among the five tested fungi, DMSO alone has shown PIMG higher than 50% in *A. alternata* and *C. gloeosporioides*. Therefore, only PJ-WS-LE solution was used for the rest of the investigations, being dissolved in distilled-water insure that antimicrobial activity is related to active phytochemicals only. PDA plates with 20 mg/ml of PJ-WS-LE extract showed 100% inhibition of the growth of *B. cinerea* and *A. alternata* which are two major food spoiling fungi. High PIMG were shown also against *G. candidum* (66.2%), *C. gloeosporioides* (60.3%) and *F. oxysporum* (59.2%). T-test showed significant difference between the mycelium diameters of the controls and the experimental plates for all fungal strains (P < 0.05) except for *A. niger* (P = 0.279). Pure PDA plates were used as negative control and PDA plates with Clotrimazole were used as positive control plates with zero fungal growth. All trials were conducted in four replications for each fungal species, average mycelium diameter ± standard error (SE) of each fungus in both experimental and control trials are shown in Fig. 2.

##### Pour plate method

*P. citrinum* was 100% inhibited upon soaking its spores for 1.5 h in 20 mg/ml of the PJ-WS-LE extract followed by pouring them in PDA plates of the same PJ-WS-LE extract concentration. As for the case of *P. chrysogenum*, the fungal strain was not affected by the extract.

#### Antibacterial effect

**Figure 3:**
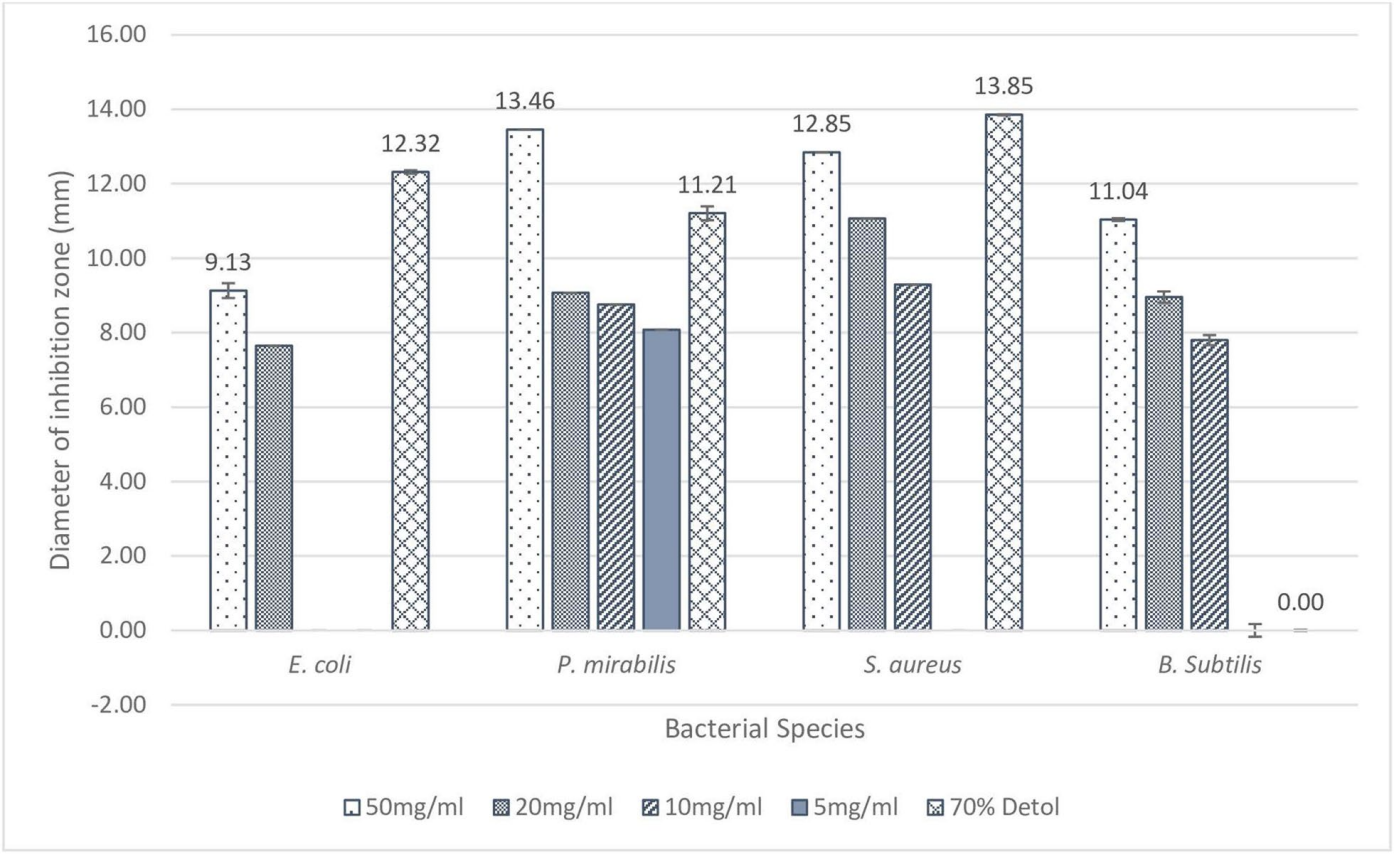
Diameter of the inhibition zone (with standard deviation bars, N = 3) of different concentrations of PJ-WS-LE extract against four bacterial strains using disk diffusion method and upon incubation at 37 °C for 24 h. ^abcde^Within each bacterial species, treatment columns with different letters have their values significantly different as shown by Tukey’s test at *p* ≤ 0.05.

Disk diffusion method was used to characterize the antimicrobial effect of the crude extracts on four bacterial isolates: *Escherichia coli*, *Proteus mirabilis*, *S. aureus,* and *B. subtilis*. PJ-WS-LE extract showed the best efficacy, its various concentrations showed good inhibition of the four tested strains as summarized in Fig. 3. All bacteria showed a dose-dependent response against the extract with the largest inhibition zone with 50 mg/ml PJ-WS-LE extract.

The antibiogram showed that the bacterial strains tested were multi-drug resistant (Table 2). All disk diffusion results were conducted in triplicates.

**Table 2:** Antibiogram of four bacterial strains to (Ampicillin (AMP), Amoxicillin (AMX), Bacitracin (B), Carbenicillin (CB), and Cephalothin (CR)) (S: susceptible, R: resistant, I: intermediate).

	AMP	AMX	B	CB	CR
*E. coli*	22.6 S	21.75 I	0 R	25.1 I	0 R
*P. microbilis*	28.3 S	29.9 S	0 R	30.97 S	20.75 S
*S. aureus*	9.36 R	9.03 R	0 R	12.57 R	16.62 I
*B. subtilis*	10.64 R	10.2 R	8.39 I	10.26 R	28.15 S

#### Anti-yeast effect

The effect of PJ-WS-LE extract against *C. albicans* was tested using the disk diffusion method. Dose-dependent inhibition of the yeast strain was very clear with the highest diameter of inhibition (18.43 mm) shown with 50 mg/ml of the extract which is very close to the diameter of inhibition zone in the positive control (22.5 mm).

### Extraction yield

Among the tested extracts, PJ-WS-LE extract was the most effective as an antimicrobial agent, therefore, the extraction method yield was calculated each time new extract was prepared. The average extraction yield was around 11%.

### PJ-WS-LE extract stability

**Figure 4:**
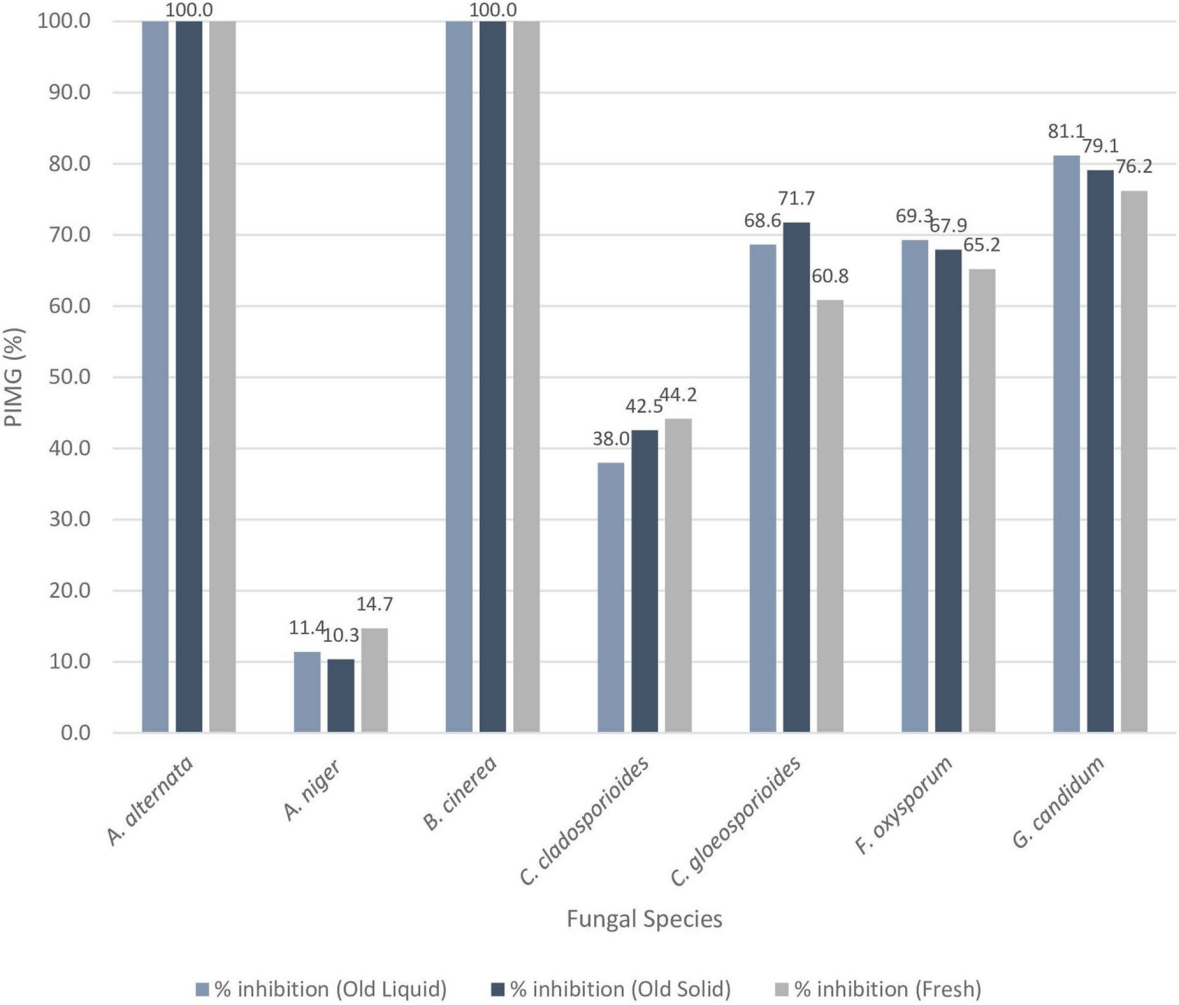
Percent inhibition mycelial growth of seven fungal strains by 20 mg/ml of 6-month-old PJ-WS-LE extract preserved as dry powder and as aqueous solution compared to fresh extract.

Agar diffusion method was conducted using the seven previously tested fungal strains. Results confirmed stability of the active antifungal compounds of the 6-month-old crude extract preserved at 4 °C in powder form and in aqueous solution form. Figure 4 shows the PIMG of the seven fungal strains using 6-month-old extract compared to freshly prepared PJ-WS-LE extract. T-test showed no significant difference was observed between the three experimental batches (p > 0.05).

### Determination of minimum inhibitory concentration

Table 3 shows the different MICs of the PJ-WS-LE extract to all tested fungi, bacteria, and yeast isolates. Results were read after 72 h of incubation. Spores that germinated at all extract concentrations had their MICs valued as > 50 mg/ml. Besides the three fungal species that showed total inhibition of their growth on PDA plates (*B. cinerea*, *A. alternata*, and *P. citrinum*), *C. cladosporioides* and *G. candidum* had their spore germination totally inhibited in the presence of the extract. Interestingly, *A. niger* had an MIC of 2.5 mg/ml at 48 h, yet spores grew in all wells at 72 h which means that the extract slowed down the fungal growth.

**Table 3:** Minimum inhibitory concentrations of PJ-WS-LE extract against different microorganisms.

Fungal species	MIC (mg/ml)	Bacterial Type	MIC (mg/ml)
*A. niger*	> 50	*B. subtilis*	0.125
*P. chrysogenum*	> 50	*S. aureus*	0.5
*B. cinerea*	1	*E. coli*	> 50
*F. oxysporum*	> 50	*P. mirabilis*	> 50
*A. alternata*	1		
*P. citrinus*	2		
*C. gloeosporioides*	> 50		
*C. cladosporioides*	4		
*G. candidum*	2.5		
*C. albicans*	0.5		

As for bacterial strains, it is worth mentioning that the extract was able to completely kill gram-positive strains in liquid culture even at very low concentrations, while gram-negative strains showed cellular activity at all extract concentrations even though they were partially inhibited on agar.

### Crude extract effect on fungal spore germination

Spore suspensions exposed to various concentrations of PJ-WS-LE extract for 24 h with shaking at 25 °C were evaluated for germination percentage. Results are summarized in Table 4. Percentages of spore germination at the start of the experiment (before incubation) were also calculated and subtracted from the final percentages of spores germinating in the presence of the leaf extract to rule out spores that were already germinated on the PDA plates. This explains the negative values of *A. alternata* and *G. candidum*. The extract concentration which led to the maximum inhibition of spore germination was 8 mg/ml for all fungal strains that showed total inhibition in the 96-well plate experiment except for *C. cladosporioides*. *C. gloeosporioides* and *P. chrysogenum* showed a dose-dependent spore germination inhibition when exposed to the leaf extract. The most resistant fungal species was *F. oxysporum*, in which spore germination only decreased by 8.9% in the presence of 8 mg/ml of the extract. Finally, microscopic observation showed that even the most resistant strains of germinated spores exhibited stress-like symptoms.

**Table 4:** Percentage of germinating spores after 24 h of exposure to different concentrations of PJ-WS-LE extract at 25 °C with shaking (150 rpm).

	Control	2 mg/ml	4 mg/ml	8 mg/ml
*A. niger*	44.93	17.81	9.38	4.67
*P. chrysogenum*	95.74	40.46	30.43	21.70
*B. cinerea*	79.21	0.00	0.00	0.00
*F. oxysporum*	99.00	99.00	99.00	89.91
*A. alternata*	52.96	6.94	3.87	− 0.89
*P. citrinus*	45.15	47.46	24.16	4.58
*C. gloeosporioides*	59.31	26.87	19.81	14.13
*C. cladosporioides*	51.79	56.56	48.51	49.61
*G. candidum*	72.06	75.29	16.28	− 0.72

### Mode of action of fungal or bacterial inhibition

**Figure 5:**
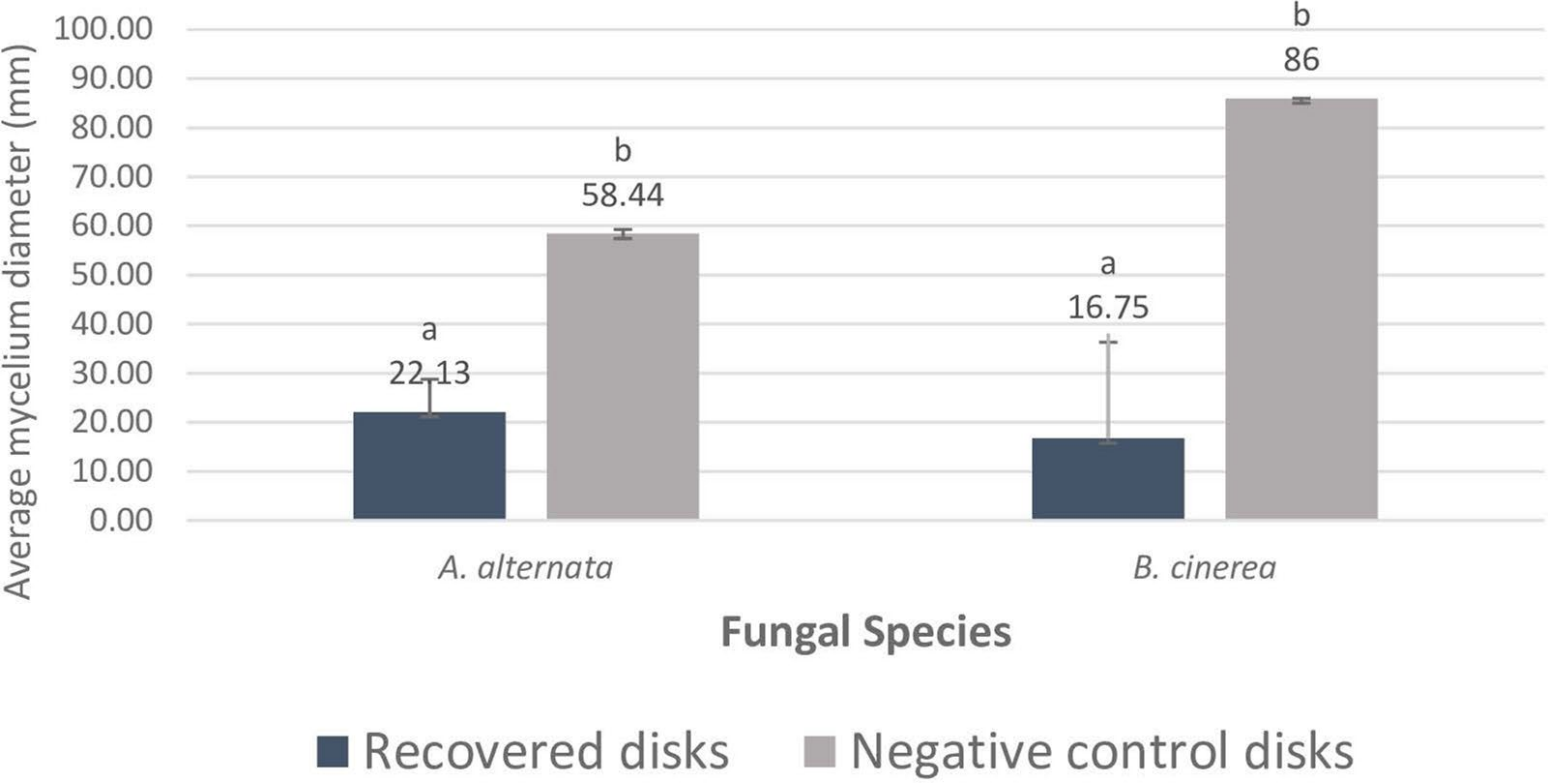
Average diameter ± SE of fungal plugs transferred from 20 mg/ml PJ-WS-LE extract plates to clean PDA plates compared to direct fungal plugs growth on clear PDA plates. ^ab^Treatment columns with different letters have their values significantly different as shown by t-test for data of fungal species.

When fungal mycelia that failed to grow in the presence of the plant extract were transferred to fresh PDA plates, the 7 days-old results showed an average diameter of *A. alternata* and *B. cinerea* much lower in recovered disks than in the negative control. Some of the replicate disks could not re-grow which led to a high standard error (Fig. 5).

When fungal spores and bacteria were incubated in the presence of PJ-WS-LE extract for 48 h (8 mg/ml) and then spread on clean media, results showed that the mode of inhibition of *A. alternate*, *B. cinerea* and *C. albicans* is fungicidal, while the mode of inhibition of *G. candidum* and *C. cladosporioides* is fungistatic. As for the effect of the leaf extract on *S. aureus* and *B. subtilis*, results showed bacteriostatic mode of action.

### The effect of PJ-WS-LE extract on the microscopic morphology of the studied fungal species hyphae and spores

**Figure 6:**
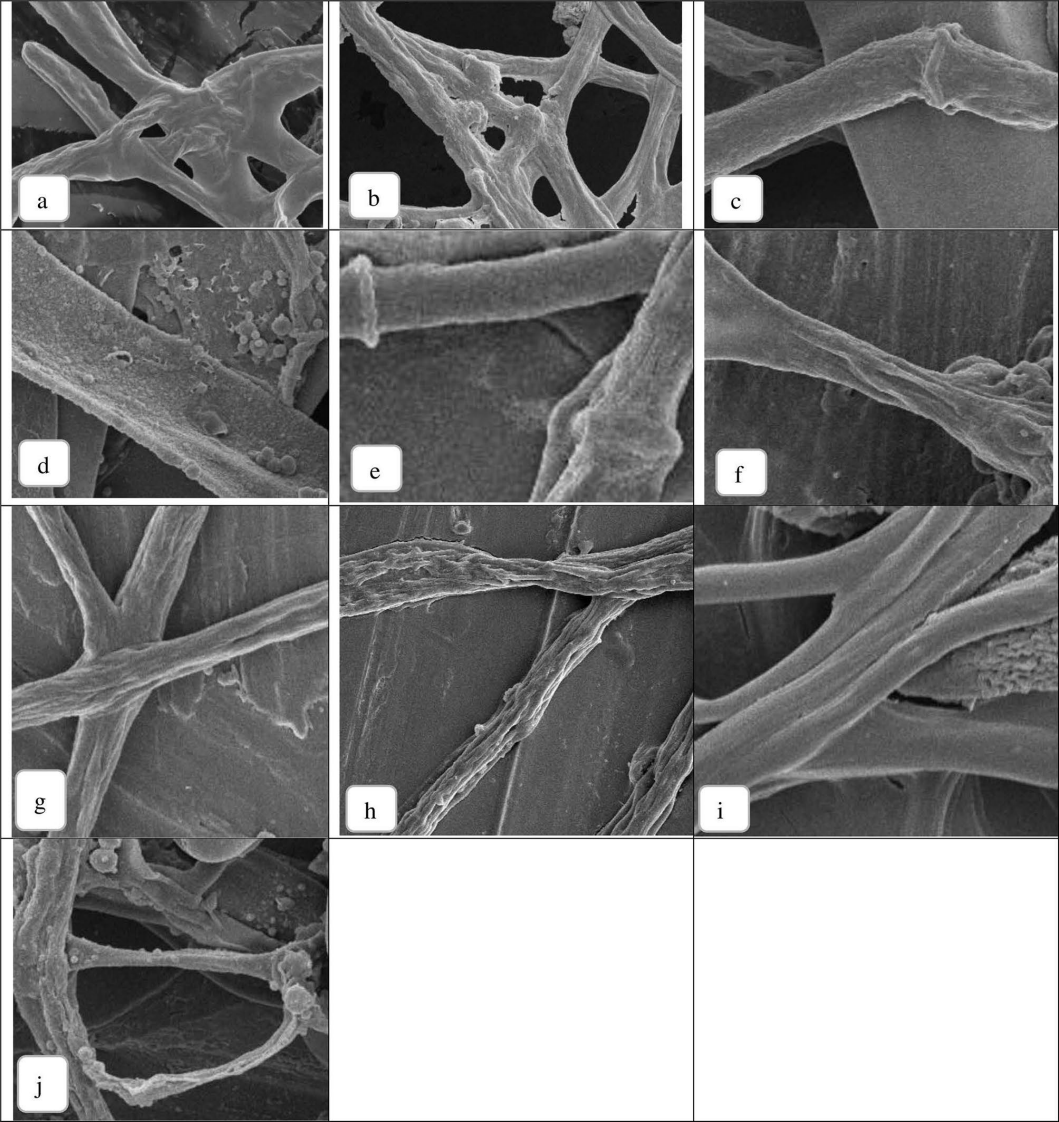
SEM images of the hyphae of control fungi and fungi treated with 8 mg/ml PJ-WS-LE extract**.** *A. niger*: non-treated (**a**) and treated (**b**) (5.000×). *B. cinerea*: non-treated (**c**) and treated (**d**) (5.000×). *G. candidum*: non-treated (**e**) and treated (**f**) (5.000×). *C. gloeosporioides*: non-treated (**g**) and treated (**h**) (10.000×). *A. alternata*: non-treated (**i**) and treated (**j**) (5.000×).

**Figure 7:**
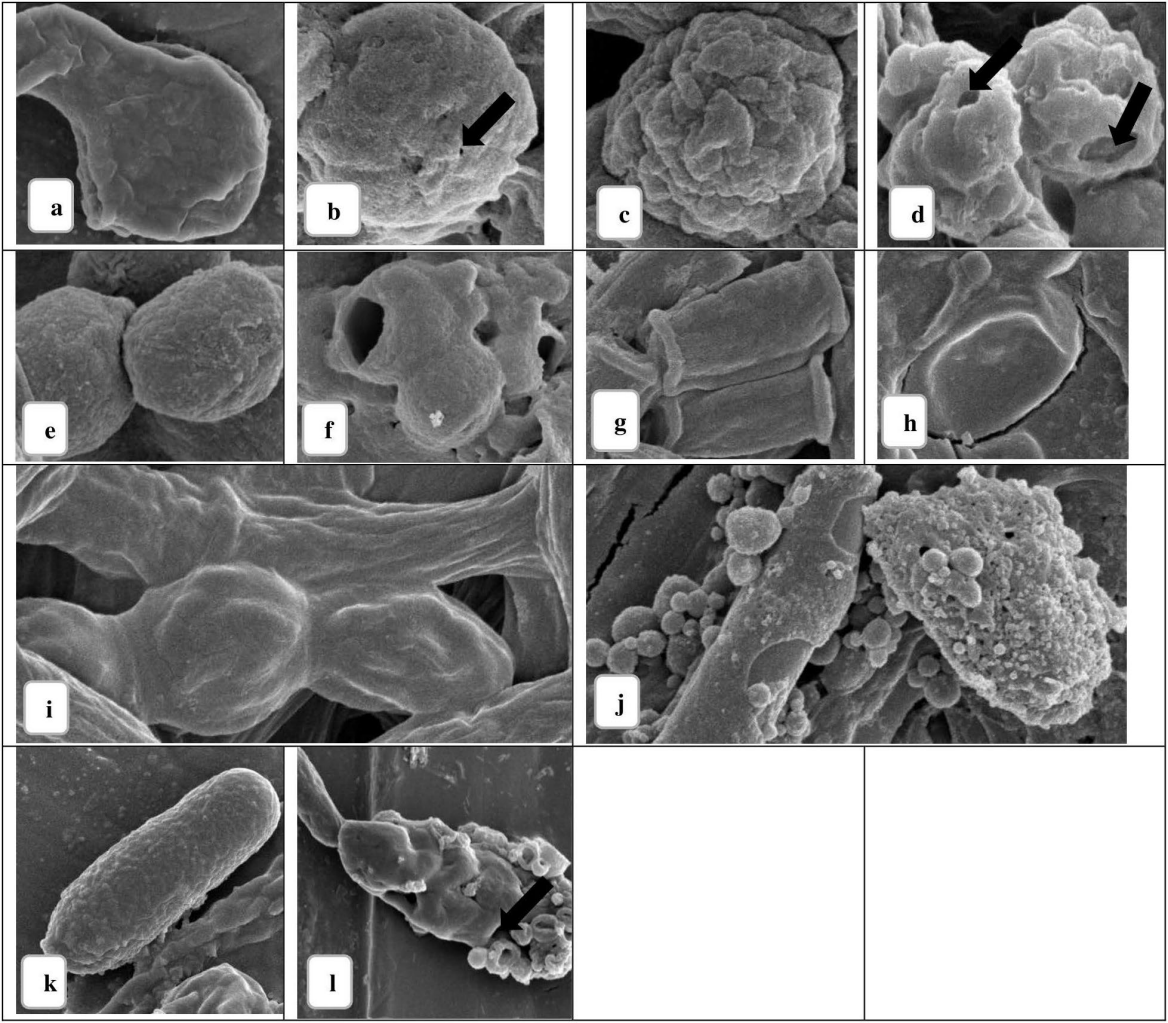
SEM images of the spores of control fungi and fungi treated with 8 mg/ml PJ-WS-LE extract**.** *A. niger*: non-treated (**a**) and treated (**b**) (25.000 ×). *P. citrinum*: non-treated (**c**) and treated (**d**) (25.000×). *C. cladosporioides*: non-treated (**e**) and treated (**f**) (20.000×). *G. candidum*: non-treated (**g**) and treated (**h**) (10.000×). *F. oxysporum*: non-treated (**i**) and treated (**j**). *C. gloeosporioides*: non-treated (**k**) and treated (l) (10.000×).

SEM was used to evaluate the effect of PJ-WS-LE extract on the hyphae and spores of nine fungal species, especially those in which growth and germination were not completely inhibited by the extract. SEM evaluation showed damage of the external morphology of all tested fungal species. *A. niger* was not entirely inhibited by the extract as shown in previous assays. However, the typical net smooth surface of the hyphal structure seen in the non-treated control samples of *A. niger* (Fig. 6a) was not maintained when it was exposed to 8 mg/ml of PJ-WS-LE extract for 24 h. The extract caused the hyphae of this fungal species to lose smoothness; exposed hyphae were more applanate with exfoliated flakes and small pores in some places (Fig. 6b). At higher magnification (25.000×), *A. niger* spores showed surface damages and small pores (arrow) (Fig. 7b) that were not seen in the control (Fig. 7a). Control (non-treated) samples of *P. citrinum* and *P. chrysogenum* showed nice filamentous tubular hyphae while treated samples showed severely fractured hyphal structure with vacuolation that might indicate leakage of essential intracellular components (data not shown). Treated *penicillium* spores were shrunken compared to control (Fig. 7c) with craters of different sizes (arrows) (Fig. 7d). *C. cladosporioides* is one of the fungi which growth was inhibited by the leaf ethanolic extract in-vitro; this was also clearly indicated by the large holes in the treated spore membranes shown by SEM (Fig. 7f), non-treated *C. cladosporioides* sample showed smooth spores (Fig. 7e). Treated *B. cinerea* mycelium lost their tubular shape (Fig. 6c) and showed degenerative changes including applanation and formation of exfoliated flakes while their spores at higher magnification (10.000×) were totally collapsed (Fig. 6d). Microscopic observation of this fungus supports the total growth inhibition observed in previous experiments.

Untreated samples of *G. candidum* showed nice tubular hyphae (Fig. 6e) that were distorted and flattened when treated with the extract (Fig. 6f). Tubular unique spores of *G. candidum* (Fig. 7g) were collapsed when treated with the antifungal extract (Fig. 7h). Spore collapse might be an indicator of loss of intracellular components as indicated earlier and therefore cell death. The growth of *F. oxysporum* was not totally inhibited by the crude extract in-vitro, yet SEM images show severe damage in both mycelium and spores. Treated *F. oxysporum* showed various levels of diastrophic and fractured mycelium and spores, in addition to vacuolation and pores in the case of treated spores (Fig. 7j) compared to normal (Fig. 7i). The treated *C. gloeosporioides* samples showed distorted mycelium with unusual surface bulges and applanation in some areas (Fig. 6h) as well as damaged spores with rugged and fractured surfaces with holes (arrow) (Fig. 7l). Figures 6g and 7k shows the normal shapes of *C. gloeosporioides* mycelium and spores for comparison. Finally, *A. alternata* is among the most affected tested fungi by PJ-WS-LE extract. Treated samples showed collapsed spores with large vacuolation while their mycelia lost their smoothness (Fig. 6i) and were shriveled and distorted with large vacuolation which also indicates loss of intracellular components that leads to death (Fig. 6j).

### Effects of PJ-WS-LE extract on grey mold and *Alternaria* rot development in artificially inoculated cherry tomatoes

**Figure 8:**
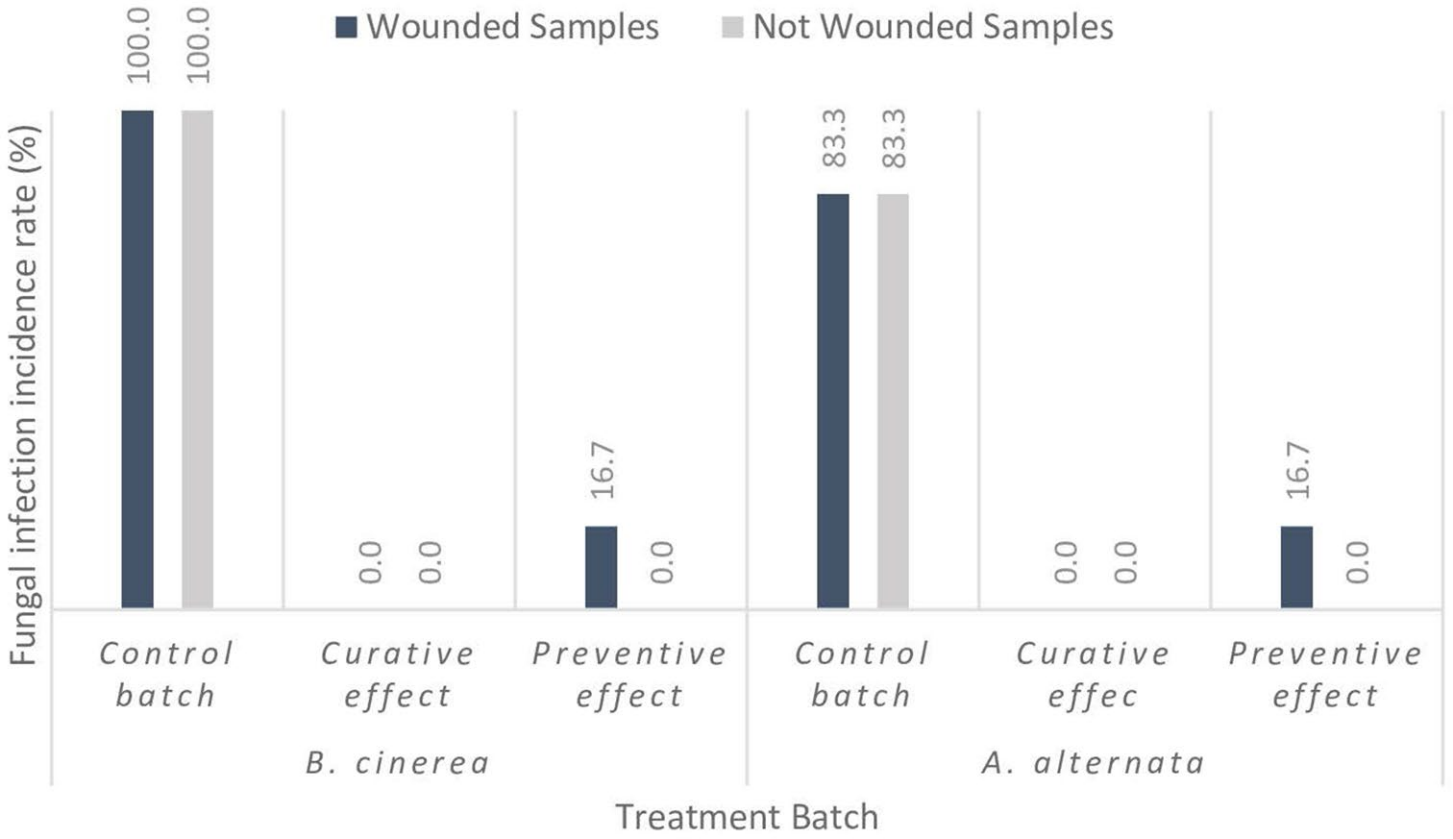
Percent infection rate of wounded and not-wounded cherry tomato samples inoculated with *A. alternata* and *B. cinerea* without PJ-WS-LE extract treatment and with PJ-WS-LE extract treatment applied to evaluate curative and preventive effects.

None of the negative control samples showed fungal growth within the two-week timeline of the experiment, which indicates the efficacy of the pre-sterilization treatment of the samples. Most of the non-treated control samples showed fungal growth, while only one treated sample out of the 24 samples inoculated with *A. alternata* showed fungal growth. Similar results were observed in the case of *B. cinerea*. Infection rate in *B. cinerea* control batches was 100% with 4.16% infection rate in the samples treated with PJ-WS-LE extract. As for the *A. alternata* control samples, infection rate was 90% while only 4.16% of the treated samples showed infection. PJ-WS-LE extract showed both curative and preventive effects on both wounded and entire samples which indicates very promising results for future market use. Figure 8 shows the details of infected samples percentage per each treatment batch. It is also worth noting that treated cherry tomato samples did not show any signs of phytotoxicity upon PJ-WS-LE extract application. Fourteen days-old samples exhibited no change in color or smell which indicates quality preservation.

## Discussion

Different parts of *P. juliflora* were evaluated for their antibacterial activity in various studies, yet studies had their gaps including the usage of organic solvents in extracts preparation and the lack of control batches for all tests. Disk diffusion method results showed that the diameters of inhibition zones of gram positive bacteria (*S. aureus* and *B. subtilis*) around PJ-WS-LE extract were higher than those caused by commonly used antimicrobials including ampicillin, Amoxicillin, Bacitracin and Carbenicillin. Singh and Verma^18^ examined the effect of 100 mg/ml of ethanolic extract of the leaves, pods and flowers of *P. juliflora* against different bacterial strains. The disk diffusion method showed that the inhibition zone diameters of *E. coli* and *S. aureus* treated with 100 mg/ml of leaf ethanolic extract were 12.81 ± 0.45 mm and 12.72 ± 0.67 mm, respectively. These numbers are close to our results in which the inhibition zone diameters of *E. coli* and *S. aureus* treated with 50 mg/ml of PJ-WS-LE extract were 9.13 ± 0.20 mm and 12.85 ± 0.01 mm, respectively. Note that half the amount of the extract prepared in this study was as effective as Singh and Verma’s extract in the case of *S. aureus. D*ifferent concentrations of *P. juliflora* leaf ethanolic extract were tested for their antimicrobial activity against different bacterial strains including *S. epidermis*, *S. aureus*, *Streptococcus spp*. *Micrococcus luteus*, *B. subtilis, Salmonella typimurium*, *Klebsiella pneumonia*, *E. coli*, and *Pseudomonas* spp. in two different studies. Results showed growth inhibition activity. A comparison between the inhibition zone diameters obtained in previous studies and the diameters obtained in our study is shown in Table 5. It is noted that *S. aureus* was more inhibited by our extract while *E. coli* and *B. subtilis* showed similar values to Sathiya and Muthuchelian’s study, while Thakur et al*.* have used higher extract concentrations. It is important to know that the novel extraction method used in this study is different than the other two studies in which leaf extract was dissolved wither in ethanol or in DMSO, which may have had antimicrobial effects themselves regardless of the phytochemcials extracted^19,20^.

**Table 5:** Diameter of inhibition zone (mm) of bacterial growth of *P. juliflora* leaf ethanolic extract as obtained in three different studies.

	*E. coli*	*S. aureus*	*B. subtilis*	*Salmonella*	*Pseudomonas*
^20^/(50 mg/ml)	9.82 ± 0.05	9.88 ± 0.68	8.74 ± 0.56	8.55 ± 0.30	8.77 ± 0.55
^19^/(100 mg/ml)	17.00 ± 0.00	6.67 ± 0.58	17.33 ± 0.58	–	16.00 ± 0.00
Current study/(50 mg/ml)	9.13 ± 0.20	11.06 ± 0.01	8.95 ± 0.15	–	–

A study by Osuru et al*.*^21^ analyzed the aqueous extract of *P. juliflora* leaves as a possible mouthwash solution, results showed that this aqueous extract inhibited the growth of *Enterococcus faecalis*, *S. aureus*, *Prevotella intermedia*, *Porphyromonas gingivalis* and *Aggregatibacter actinomycetemcomitans.* Note that the inhibition zone diameter of *S. aureus* was found to be 10.16 ± 0.28 mm, which is still lower than the inhibition zone diameter obtained with the extraction method used in our study (11.06 ± 0.01), note that both extraction methods involved water as a final solvent of the extracts^21^.

A recent study examined the enhanced effect of *P. juliflora* methanolic extract combined with silver nanoparticles as an antimicrobial agent against strains of *Candida* sp. and *Staphylococcus* sp. Leaves of *P. juliflora* were dried, grinded, and soaked in methanol for 24 h, and the extract was filtered and mixed with a solution of silver nitrate (AgNO_3_). Upon color change, the precipitate was washed, dried, and used for further analysis. Results showed that 1 µg/ml of this nano-powder was capable of totally inhibiting the growth of *MRSA*, *C. albicans* and C. *tropicalis*^22^. Note that in our study, both *S. aureus* and *C. albicans* were completely inhibited by our extract with an MIC of 500 µg/ml. The combined effect of *P. juliflora* leaf aqueous extract with silver nanoparticles encapsulated in chitosan showed a higher diameter of inhibition zone (22 mm) against *E. coli* when compared to the current study (9.13 mm), this is due to the combined antimicrobial effect of chitosan and the synthesized nanoparticles^23^. Zinc monoxide nanoparticles derived from *P. juliflora* leaf aqueous extract had also a successful antimicrobial effect against *E. coli* and *B. subtilis* with an inhibition zone diameter of 23 mm and 19 mm, respectively, at concentrations of 100 µg/ml^24^. These findings have shown that plant extracts enhanced with nanoparticles or coating material lead to a higher antibacterial efficacy. However, nanoparticles enhanced studies lacked the control batches that test for the antimicrobial effect of the metals nanoparticles themselves. Unlike PJ-WS-LE extract where phytochemicals were dissolved in water, these materials require further analysis to test for their possible health risks and to ensure that they are safe for the environment if they are to be used in the field or as a postharvest controller.

Various preliminary studies showed also the antifungal effect of various extracts of *P. juliflora*. A study by Raghavendra et al*.*^25^ showed that the aqueous leaf extract of *P. juliflora* significantly decreased the mycelial growth of *A. alternata* isolated from tobacco leaves with a PIMG of 71.59% at a concentration of 24%. This number is comparable to our results in which the PIMG of *A. alternata* was found to be 80.57% after exposure to 2 mg/ml of PJ-WS-LE extract and 100% after exposure to 20 mg/ml of the extract. On the other hand, a study, which examined the effect of the methanolic extract of *P. juliflora* leaves on the soil-borne pathogenic fungus *Sclerotium rolfsii* did not find any significant antifungal activity against this species^26^. Bazie et al.^27^ tested the antifungal effect of leaf extracts from different plant species against *Colletotrichum musae*, the causative agent of postharvest banana anthracnose, and they found that the methanolic extract of *P. juliflora* leaves showed the best results in fungal growth inhibition followed by *Acacia albida*. A study in 2009 examined the antifungal effect of leaf extracts from multiple plant species against different *Fusarium* species found that using a fresh solution of *P. juliflora* leaves resulted in a PIMG of 80.25% against a species of *F. oxysporum*, compared to 59.2% obtained in our study. Note that the extraction methods including the final solvent and the concentrations used in the two studies are different^11^. More recent study showed that using fresh extract from the maceration of *P. juliflora* leaves had a low antifungal efficacy against *Alternaria solani*, with a PIMG of 27.14% at a 10% concentration, while our extraction method showed a very high antifungal efficacy (100%) against a strain of Alternaria (*A. alternata*)^28^.

Many studies aimed to find the MIC of *P. juliflora* crude extracts, enriched extracts, and purified compounds against a scattered group of microorganisms, however, none of the studies was comprehensive enough to test for the efficacy of a particular extract against a range of spoiling organisms. Santos et al.^29^ subjected the ethanolic extract of *P. juliflora* pods to acid–base treatments to obtain alkaloid-enriched extracts. The ethanolic extract in that study was suspended in acetic acid solution, and then the aqueous phase was extracted with chloroform or ethyl acetate at different pH levels. The basic chloroformic extract showed the best antimicrobial efficacy as well as a high alkaloid concentration. The MIC of our PJ-WS-LE extract on *S. aureus* and *B. subtilis* were 0.5 mg/ml and 0.125 mg/ml respectively, while the MICs obtained using the alkaloid enriched extract, particularly the basic chloroformic extract, were 0.05 mg/ml for *S. aureus* and above 0.1 mg/ml for *B. subtilis*. It is worth noting that crude extracts could be safer for human health and for the environment than concentrated phytochemicals which might exhibit toxicity. An older study by Singh and Verma^18^ found that the MIC of the alkaloid rich fraction of the ethanolic extract of *P. juliflora* leaves on strains of *S. aureus* and *B. cereus* was 0.05 mg/ml, compared to our MICs of similar strains of *S. aureus* and *B. subtilis* (0.5 mg/ml and 0.125 mg/ml). The lower MIC results from Singh and Verma’s research can be justified by the concentrated active phytochemicals in the alkaloid-rich fraction**.** Both previously discussed studies showed lower MICs compared to our study because the use of concentrated active compounds normally result in lower MICs than the usage of crude extracts. However, if crude extracts continue to demonstrate successful inhibitory activity against microorganisms, they will be of commercial interest as they have feasible extraction methods that can be conducted in developing nations at a low cost. In addition, crude extracts are likely to have lower toxicity compared to concentrated pure phytochemicals^30^. The investigation of another species of *Prosopis* showed that the aqueous ethanolic extract of stem bark of *Prosopis chilensis*, which are rich with tannins, has significant antimicrobial activity against *Micrococcus luteus*, *Bacillus Subtilis*, *Bacillus cereus*, *Staphylococcus aureus*, *Streptococcus pneumonia*, and *Cryptococcus albidus*. The MICs of the stem bark extract against *B. subtilis* and *S. aureus* were 0.16 mg/ml and 0.62 mg/ml respectively, which are very close to the MICs of our PJ-WS-LE extract (0.125 mg/ml and 0.5 mg/ml)^31^. The similarity between these results suggests the presence of common active phytochemicals in the two extracts. Alkaloid extracts of *P. juliflora* showed a complete inhibition of *A. alternata* growth at a concentration of 1 mg/ml while common fungicides in the market are typically used at a concentration of 2 mg/ml. This number is equal to the MIC of our crude extract against *A. alternata*^25^.

Spores germination experiment showed no significant difference in the spore germination of *C. cladosporioides* in the presence or absence of the extract, which was not consistent with the previous experiment results. It is very possible that shaking allowed the fungi to escape the extract inhibition mechanism. It is worth noting also that *A. niger* showed a very low level of spore germination (4.67%) in the presence of 8 mg/ml of the extract within 24 h. This was consistent with previous results which showed that *A. niger* growth is delayed when exposed to the extract and those results might change if incubation time is increased. Our research showed that PJ-WS-LE extract did not completely inhibit the fungal growth and spore germination of *A. niger*. However, the smooth hyphal network of this *Aspergillus* strain was not maintained when it was exposed to 8 mg/ml of the extract for 24 h. Similarly, the treatment of *Aspergillus ochraceus* with 3-carene, a component of *Melaleuca alternifolia* oil, did not fully inhibit fungal growth, yet it damaged the external morphology of hyphae and spores as observed using SEM^32^. Such damages might play a role in decreasing fungi pathogenesis in-vivo.

*B. cinerea* treated with PJ-WS-LE extract resulted in completely collapsed spores and degenerative changes in their mycelium that could be due to a decrease in exopolysaccharide (EPS) formation in their outer membrane. Similar results were observed when *B. cinerea* were treated with phenazine-1-carboxylic acid (PCA) produced by the *Pseudomonas aeruginosa* LV strain. Hyphae of the treated fungi lost smoothness and formed unusual surface bulges^33^. The growth of *F. oxysporum* was not inhibited by our crude extract *in-vitro*, yet SEM images showed severe damage in mycelium and spores of both tested strains. Similarly, SEC imagery of *Fusarium sporotrichioides* treated with *Mentha piperita* essential oil showed distorted and shrunken mycelia compared to the control^34^. Another study conducted on an economically important pathogenic *Fusarium* strain known as *Fusarium verticillioides* also showed slender, shrunken, and winding hyphae that lost their linearity with some depressions on the surface^35^.

Various antifungal agents cause morphological changes similar to those caused by PJ-WS-LE extract. When the two pathogenic fungi *Mycrosporum gypseum* and *Trychophyton mentagrophytes* were treated with the lyophilisate of granular gland secretion from *Duttaphrynus melanostictus* frogs, the fungal cells showed cellular deformations and pores. Hyphae of *M. gypseum* showed shrinkage while those of *T. mentagrophytes* showed shrinkage and pores^36^. Recently, the treatment of *Villosiclava viren*s, an emerging disease of rice panicles, with the essential oils of 18 plants showed promising results with cinnamon bark oil, cinnamon oil and *trans*-cinnamaldehyde. Scanning electron microscopic imaging of *V. viren*s treated with the vapor of one of the effective essential oils exhibited degenerative changes in the hyphal morphology including exfoliated flakes, applanation, vacuolation and shriveling. Treatment with direct contact of essential oils caused more severe exfoliated flake damage with collapse and blistering^37^. The morphological changes caused by PJ-WS-LE extract are indicators or stressed fungi which might lose their pathogenicity against fresh produce with time.

The *in-vivo* preservative effect of PJ-WS-LE extract on cherry tomatoes was comparable to the effect of low fermenting yeast (*Lachancea thermotolerans*) volatile organic compounds (VOCs) on artificial *F. oxysporum* infection. The assay showed that 76% of the control cherry tomatoes were infected while none of the treated batches showed any infection^38^. Zeidan et al. results are similar to those of PJ_WS_LE extract that showed 100% *B. cinerea* infection and 90% *A. alternata* infection in the control batches with only 4.16% of the treated batches showing infections in each of the evaluated fungal strains. However, the application of liquid solution such as PJ-WS-LE extract in field or as post-harvest diseases controller could be more practical.

PJ-WS-LE extract has a novel, safe and affordable extraction method that makes it a promising anti-spoiling agents, the growth of major spoiling agents was totally inhibited in its presence. All tested spoiling agents showed stress-like symptoms under the SEM when they were exposed to low concentrations of the extract. *In-vivo* assay showed a successful protection of infected fruits against fungal spoilage and hyphae development. Future studies on the revealed extract would include large scale in-vivo analysis and fractionation work to identify active phytochemicals, the additional studies are needed to further prove the extract efficacy for future promotion as a replacement for anti-spoilage chemicals used in the market.

## Materials and methods

### *Prosopis juliflora* samples collection and extracts preparation

*Prosopis juliflora* leaves and plants were collected from healthy mature plants from the fields of Qatar University Campus after the proper permission. All methods were carried out in accordance with relevant guidelines and regulations. The plant status in Qatar is considered as exotic and invasive species. Leaves and fruits were washed thoroughly with tap water followed by sterilized distilled water and they were left to dry in a sterilized oven at 45 °C. Dried leaves and fruits were grinded to powder state and then stored at 4 °C until extract preparation^39^. Twenty grams of leaf or fruits powder was mixed with 200 ml of sterile distilled water for aqueous extract or 70% ethanol for ethanolic extract, bottles were then transferred to a water bath at 45 °C with shaking (50 cycles/min) for 48 h. Note that in aqueous extracts preparation the bottles were pre-heated to 70 °C for 3 h before incubating at 45 °C. Extracted liquid was centrifuged at 4500 rpm for 5 min^40^. Supernatant was filtered using Whatman filter papers and poured into 150 mm glass Petri plates to be transferred to a pre-sterilized oven at 45 °C to evaporate the solvent. The dried (powdery or gummy) material was aseptically scratched, weighed and stored at 4 °C. A second elution of 70% ethanol was also prepared^16,26^.

**Figure 9:**
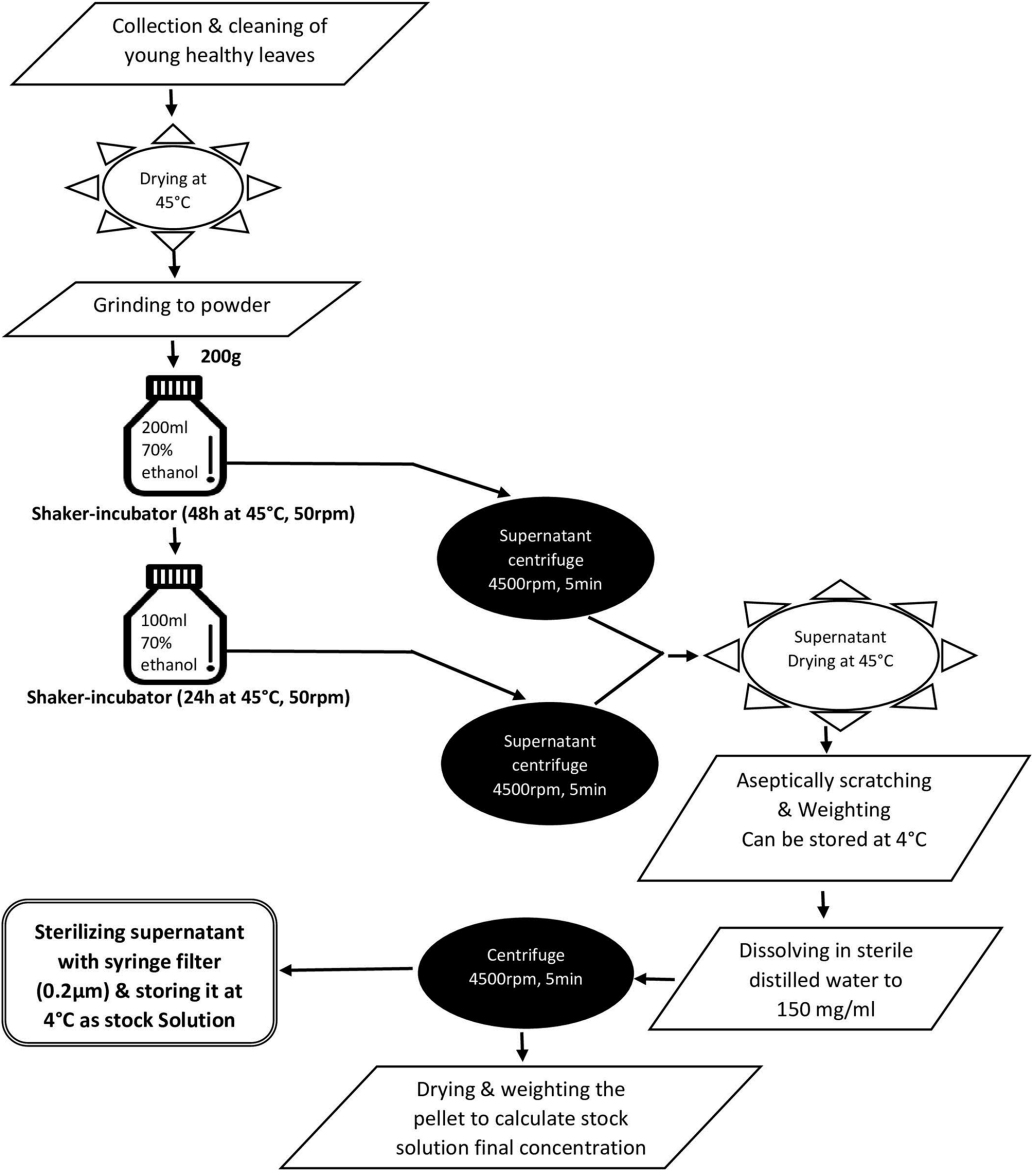
Flow diagram of PJ-WS-LE extract preparation.

Ethanolic extracts were dissolved in: (a) 4% DMSO to completely dissolve the extracted material into a homogeneous solution, (b) sterile distilled water to prepare a suspension of 150 mg/ml. Tubes of the suspension were centrifuged and the water-soluble supernatant was taken and stored at 4 °C to be used in the analysis. The water-insoluble pellet was incubated at 45 °C to dry. The pellet was then weighed, and the new concentration of the solution was re-calculated. It was noticed that the mass of the pellet was around 9.5% of the initial total mass of the ethanolic extracted powder which makes the final concentration of *P. juliflora* water-soluble leaf ethanolic (PJ-WS-LE) extract around 125 mg/ml. All suspensions were sterilized using syringe filter 0.2 µm. Figure 9 represents the steps of preparation of PJ-WS-LE extract. Extraction method yield of PJ-WS-LE extract was also determined as follows^41^:

$$ {\text{Extraction yield}} = \frac{{{\text{Mass of extracted powder}} \times 100}}{{\text{Mass of leaves powder used}}}. $$

### Food spoiling microbial isolates

The antimicrobial in-vitro efficacy of *P. juliflora* water-soluble leaf ethanolic (PJ-WS-LE) extract was tested against bacterial, fungal and yeast strains that are known to cause food contaminations. Bacterial and yeast strains taken from the laboratory collection previously isolated and identified or purchased, the collection is currently stored at Qatar University, Department of biological and environmental sciences, chosen microbial types include: *E. coli* ATCC 11775, *Bacillus*, *S. aureus* ATCC BAA976, *P. microbilis* and *C. albicans*. The fungal stains used were isolated and microscopically identified in a previous study (Table 6)^42^.

**Table 6:** Fungal strains used in the in-vitro analysis study.

Fungi	Origin	Country of origin
*Alternaria* sp.	Cucumber	Qatar
*Aspergillus* sp.	Cucumber	Lebanon
*Botrytis* sp.	Tomato	Lebanon
*Cladosporium* sp.	Tomato	Morocco
*Colletotrichum* sp.	Orange	Morocco
*Geotrichum* sp*.*	Tomato	Netherland
*Fusarium* sp.	Cucumber	Qatar
*Penicillium* sp. 1	Orange	Australia
*Penicillium* sp. 2	Cucumber	Qatar

The fungal strains had their DNA extracted using the DNeasy Plant Mini Kit (QIAGEN, Germany) according to the manufacturer protocol. The Internal Transcribed Spacer (ITS) regions of fungal ribosomal DNA (rDNA) was amplified by PCR using ITS1 and ITS 4 primers. PCR products were purified using Invitrogen Quick PCR Purification Kit (Thermo Fisher Scientific, US) as indicated by the manufacturer. Sanger Sequencing was used to sequence the PCR products. BioEdit software was used to read the sequencing results. Sequences of DNA fragments were used in fungal species identification using NCBI-BLAST^43^.

### Agar diffusion method for antifungal activity evaluation

The antifungal effects of the prepared extracts were tested against *A. alternata*, *B. cinerea*, *F. oxysporum, A. niger, C. gloeosporioides, C. cladosporioides* and *G. candidum*. Potato dextrose agar (PDA) (Difco-USA) plates of 20 mg/ml of aqueous or ethanolic extracts of *P. juliflora* leaves or fruits were prepared by adding the appropriate volume of stock solutions previously prepared to 50 °C PDA media^26^, both ethanolic extract stock solutions dissolved in sterile distilled water and in 4% DMSO were tested. Once the media solidified, a plug of 6 mm of agar was aseptically removed from the center of each Petri dish. Plugs of four-day-old fungi culture of the same size were transferred aseptically to the experimental plate. The experiment was run in four replications. Plates with 1 ml of Clotrimazole per plates (10 mg/ml) were used as a positive control. Pure PDA plates and PDA plates with 4% DMSO were also inoculated and used as a negative control. All plates were incubated at 25 °C for 5 days. Percent inhibition of mycelial growth (PIMG) was then calculated using the formula below. Mean diameter mycelial growth was determined by measuring the diameter of the infected area in two perpendicular directions^33^.

$$ {\text{PIMG}} = \frac{{\left( {{\text{dc}} - {\text{dt}}} \right) \times 100}}{{{\text{dc}}}}. $$

dc and dt are the average mycelium diameter in the negative control plate and in the treated plate, respectively^5^.

### Antibacterial activity assay

The disk diffusion method was used to characterize the antimicrobial effect of aqueous and ethanolic extracts of *P. juliflora* leaves and fruits on bacterial (*E. coli*, *S. aureus*, *B. subtilis*, and *P. microbilis*) and yeast (*C. albicans*) isolates. A microbial lawn of each isolate was spread on Mueller–Hinton agar (HiMedia-India). Sterile paper disks (Thermo Scientific Oxoid Blank Antimicrobial Susceptibility Disks) of 5 mm diameter were inoculated with 20 µl of different concentrations of each extracts (1, 5, 10, 20, and 50 mg/ml). Dry disks were then transferred with sterile forceps into the inoculated agar plates. Disks with sterile distilled water were used as negative controls and antibiotic disks were used as positive controls. The antibiogram of the bacterial isolates were also determined to compare the effect of PJ-WS-LE extract to commonly used antibiotics (Ampicillin (AMP), Amoxicillin (AMX), Bacitracin (B), Carbenicillin (CB), and Cephalothin (CR))(Oxoid-USA). Plates were incubated at 37 °C for 24 h. The experiment was conducted in triplicates^44^.

### Preliminary results evaluation

Among the leaves and fruits, aqueous and ethanolic extract, only the extract(s) with good preliminary results in the antifungal and antibacterial assays was (were) selected to be further tested. In addition, only the solvent that shows no antimicrobial activity on its own was further used.

### Pour plate method

The pour plate method was used to test the effect of PJ-WS-LE extract against *Penicillium* strains. Heamatocytometer was used to prepare spores suspension of 3 × 10^4^ spores/ml, then a serial dilution was prepared to reach a suspension of 30 spores/ml which was used for inoculation. One milliliter of the final suspension was first incubated for 90 min at 25 °C in 20 mg/ml of PJ-WS-LE extract to increase contact time between the spores and the extract. Spores were then inoculated into PDA plates of 20 mg/ml of PJ-WS-LE extract. Percent colony growth inhibition was later calculated.

$$ {\text{Percent colony growth inhibition}} = \frac{{\left( {{\text{Nc}} - {\text{Nt}}} \right) \times 100}}{{{\text{Nc}}}}. $$

Nc and Nt are the number of colonies growing in the control plate and in the treated plate, respectively.

### PJ-WS-LE extract stability

Powder and liquid extracts were stored at 4 °C for 6 months. Their antifungal activity against the previously tested seven fungal strains was re-evaluated using agar diffusion method to determine active phytochemical stability.

### Determination of minimum inhibitory concentration

Minimum inhibitory concentrations (MIC) of PJ-WS-LE extract against all previously tested microorganisms were determined using 96-well plate. Each well was inoculated with 100 μl of nutrient broth (Difco-USA) for bacterial MIC determination and 100 μl of potato dextrose broth (PDB) (HIMEDIA-India) for fungi and yeast MIC determination. Twenty different concentrations of PJ-WS-LE extract were evaluated (from 50 to 0.125 mg/ml). Wells were inoculated with the various microorganism suspensions, spore suspensions of 10^4^ spores/ml were used. All concentrations were tested in four replications. Clear media was used as a negative control. Wells with no microbial suspensions were also used as control trials. Fungicide (1% Clotrimazole) and antibiotics (7.5 mg/ml Ampicillin) were used as positive controls for fungal and bacterial testing, respectively. Resazurin (HIMEDIA-India) was added in all wells to monitor bacterial and fungal growth. Resazurin is a blue dye that becomes pink and fluorescent upon cellular activity^45^. Resazurin stock solution was prepared by dissolving 0.27 g of Resazurin powder in 40 ml of sterile distilled water. Minimum inhibitory concentration (MIC) of PJ-WS-LE extract to various microorganisms was observed and recorded. The MIC is the lowest concentration that does not show a change of Resazurin color within the incubation time^46^. Well plates were incubated at 25 °C and results were recorded at 72 h incubation period.

### Crude extract effect on fungal spore germination

Spores suspensions of the nine molecularly identified fungal strains were inoculated into tubes of different concentrations of PJ-WS-LE extract around the fungi MIC (2, 4, and 8 mg/ml). Negative control (no extract) tubes were also prepared. The experiment was conducted in triplicates following the experimental design in Table 7.

**Table 7:** Spores germination experimental design.

	Negative control	2 mg/ml (µl)	4 mg/ml (µl)	8 mg/ml (µl)
PDB	1700 µl	1668	1636	1573
LE extract of 125 mg/ml stock	0	31.7	63.5	127
Spores suspension	300 µl	300	300	300
Resazurin	50 µl	50	50	50

Test tubes were incubated at 25 °C with 150 rpm shaking for 18 h. Slides were prepared from each test tube, and conidia were stained using cotton blue (6 µl of the test tube + 6 µl cotton blue). The number of germinated conidia was counted out of 100 random conidia in three slides for each test tube and averages were calculated. A spore is considered germinated if the length of the germination tube is at least half the length of the spore itself. Slides were also prepared from the negative control tubes before incubation to find the start number of originally germinated spores. Spore germination percentages and percent inhibition of the extract were calculated as follow:

$$ {\text{Percent inhibition }}\left( {\text{\% }} \right) = \frac{{\left( {{\text{Gc}} - {\text{Gt}}} \right) \times 100}}{{{\text{Gc}}}}. $$

Gc and Gt are the germination rate in the control and treated Petri dishes, respectively^37,47^.

### Mode of action of fungal or bacterial inhibition

Microorganisms that were totally inhibited by the PJ-WS-LE extract had their mode of growth inhibition determined. PDA agar plugs were removed from the middle of clean PDA plates (without extracts) and the plugs of Fungal mycelia that had their growth inhibited by the extract were transferred to them. Plates were incubated at 25 °C for 7 days. Positive growth of the sub-culture shows fungistatic activity, while negative growth demonstrates a fungicidal effect^5^.

The mode of inhibition of PJ-WS-LE extract on bacterial and fungal spores was also determined. Spores suspension (10^4^ spores/ml) incubated in PJ-WS-LE extract (8 mg/ml) for 48 h were used to inoculate (100 µl) clean PDA plates. Control tubes without plant extract were also used to inoculate PDA plates for comparison. This inoculation was conducted using four replications. Plates were incubated at 25 °C for 5 days. As for bacteria, overnight culture of various strains were used to inoculate 1 ml of bacterial culture into nutrient broth tubes containing 10 ml of 8 mg/ml of the PJ-WS-LE extract, tubes were incubated for 48 h at 37 °C. One hundred microliter of each tube were then inoculated into clean nutrient agar (NA, Scharlau) plates. Control tubes without plant extract were also used to inoculate nutrient agar plates for comparison. This experiment was also conducted in four replications. All plates were incubated at 37 °C for 48 h.

### The effect of PJ-WS-LE extract on the microscopic morphology of the studied fungal species’ hyphae and spores

In order to check for any damages of cell structures, the hyphae and spore structures of: *A. niger*, *B. cinerea*, *G. candidum*, *C. gloeosporioides*, *A. alternata*, *P. citrinum*, *C. cladosporioides* and *F. oxysporum* were treated with PJ-WS-LE extract and examined using a scanning electron microscope (SEM). Two test tubes of each of the eight fungal species evaluated were prepared as in Table 8.

**Table 8:** Pre-setting of germination tubes used in SEM observation.

	Negative control	8 mg/ml (µl)
PDB	1700 µl	1540
LE extract of 100 mg/ml stock	0	160
Spores suspension	300 µl	300

Tubes were incubated at 25 °C with 150 rpm shaking for 24 h. Tubes were then centrifuged (5000 rpm) and the pellet was washed twice with PBS pH 7.4. Cells were fixed by re-eluting the pellet in a solution of 2.5% glutaraldehyde + 3.6% of formalin and were incubated at 4 °C for 18 h. Tubes were then centrifuged (5000 rpm) and washed three times with PBS. Pellets were dehydrated in serial ethanol dilution (25, 50, 70, 80, 90, and 100%) for 30 min for each dilution. Samples were then smeared on silver holders in thin films and left to air dry before gold coating them using Agar Sputter Coater. The SEM observations were made using Nova NanoSEM 450^32,37^.

### Effect of PJ-WS-LE extract against *A. alternata* and *B. cineraria* induced infection in cherry tomatoes

Organic locally-grown cherry tomato samples were purchased from the market and washed with disinfecting soap and water followed by sterile distilled water. Clean cherry tomato samples were dried using heat sterilized towels and categorized based on their weight ranges. Eighty four samples were divided into 14 treatment category of six samples each, samples in different categories had similar shapes and weight ranges.

Half of the cherry tomato samples were wounded, wounds were made by inserting a sterile syringe tip 5 mm deep in two opposite positions near the calyx of each sample. Two negative control treatment categories were made of sterile wounded and non-wounded plants without any treatment. *B. cineraria* control and *A. alternata* control groups are samples treated with 15 μl of spores suspension alone on the tomato calyx of wounded and non-wounded treatment categories. Spores suspension of *B. cineraria* was prepared at 10^4^ spores/ml using a heamatocytometer, while *A. alternata* spores suspension was prepared at 10^7^ spores/ml.

To test the curative effect of the extract, 15 μl of the spores suspension was applied on the calyx first and left to dry for 2 h after which the calyx was sprayed with a solution of 8 mg/ml of PJ-WS-LE extract. To test the preventive effect of the extract, the samples were first sprayed with a solution of 8 mg/ml of PJ-WS-LE extract and left for 2 h to dry then 15 μl of the spores suspension was applied. Plants were all kept in sterile paper boxes that were closed tightly and left at room temperature to have their quality and infection rate monitored on a daily basis. The experiment was ended in 2 weeks.

### Statistical analysis

Descriptive statistics data was obtained using the SPSS program (IBM SPSS Statistics 24.0). One Way ANOVA was used to calculate the significance of the effect of extract concentration at *p* ≤ 0.05 and Tukey’s test was used to separate the means at *p* ≤ 0.05. All assays were performed in four replications and results were presented in graphs showing means with standard error bars^37^.

## References

[1] Gil JG, López J, Henao-Rojas J (2020). Causes of hass avocado fruit rejection in preharvest, harvest, and packinghouse: Economic losses and associated variables. Agronomy.

[2] Cassman KG, Grassini P (2020). A global perspective on sustainable intensification research. Nat. Sustain..

[3] Mostafidi M, Sanjabi MR, Shirkhan F, Zahedi MT (2020). A review of recent trends in the development of the microbial safety of fruits and vegetables. Trends Food Sci. Technol..

[4] Basavegowda N, Patra JK, Baek K-H (2020). Essential oils and mono/bi/tri-metallic nanocomposites as alternative sources of antimicrobial agents to combat multidrug-resistant pathogenic microorganisms: An overview. Molecules.

[5] Bill M, Sivakumar D, Korsten L, Thompson AK (2014). The efficacy of combined application of edible coatings and thyme oil in inducing resistance components in avocado (*Persea americana* Mill.) against anthracnose during post-harvest storage. Crop Prot..

[6] Lukša J (2020). Fungal microbiota of sea buckthorn berries at two ripening stages and volatile profiling of potential biocontrol yeasts. Microorganisms..

[7] Mailafia S, Okoh GR, Olabode HOK, Osanupin R (2017). Isolation and identification of fungi associated with spoilt fruits vended in Gwagwalada market, Abuja, Nigeria. Vet. World.

[8] Bhalerao V, Chavan A (2020). Antifungal activity of leaf extract against mycotoxin producing fungi. Int. J. Res. Pharmaceut. Sci..

[9] Leyva Salas M (2017). Antifungal microbial agents for food biopreservation—A review. Microorganisms..

[10] Halonen N (2020). Bio-based smart materials for food packaging and sensors—A review. Front. Mater..

[11] Satish S, Mp R, Anandarao R (2009). Antifungal potentiality of some plant extracts against *Fusarium* sp.. Arch. Phytopathol. Plant Prot..

[12] Aziz MA (2018). Traditional uses of medicinal plants practiced by the indigenous communities at Mohmand Agency, FATA, Pakistan. J. Ethnobiol. Ethnomed..

[13] Henciya S (2017). Biopharmaceutical potentials of *Prosopis* spp. (Mimosaceae, Leguminosa). J. Food Drug Anal..

[14] Badri A (2017). Antioxidant activity and phytochemical screening of *Prosopis juliflora* leaves extract. Adv. Med. Plant Res..

[15] Lakshmibai R (2018). Phytochemical analysis and antioxidant activity of *Prosopis Juliflora* thorn extract. Malaya J. Biosci..

[16] Sayago JE, Ordoñez RM, Kovacevich LN, Torres S, Isla MI (2012). Antifungal activity of extracts of extremophile plants from the Argentine Puna to control citrus postharvest pathogens and green mold. Postharvest. Biol. Technol..

[17] Solanki DS (2018). Characterization of a novel seed protein of *Prosopis cineraria* showing antifungal activity. Int. J. Biol. Macromol..

[18] Singh S, Verma S (2011). Antibacterial properties of Alkaloid rich fractions obtained from various parts of *Prosopis juliflora*. Int. J. Pharm. Sci. Res..

[19] Thakur R, Singh R, Saxena P, Mani A (2014). Evaluation of antibacterial activity of *Prosopis juliflora* (SW.) DC. leaves. Afr. J. Trad. Complement. Altern. Med..

[20] Sathiya M, Muthuchelian K (2008). Investigation of phytochemical profile and antibacterial potential of ethanolic leaf extract of *Prosopis juliflora* DC. Ethnobot. Leafl..

[21] Osuru HP (2011). Comparative evaluation of the antibacterial efficacy of *P. juliflora* and three commercially available mouthrinses: An in vitro study. J. Pharm. Res..

[22] Anwar Y, Fakieh M, Ullah I, Alkenani N, Shareef M (2019). Synthesis of silver nanoparticles using *Prosopis juliflora* extract: Potential of antimicrobial and pollutants degradation performance. Desalin. Water Treat..

[23] Malini S (2020). Antibacterial, photocatalytic and biosorption activity of chitosan nanocapsules embedded with *Prosopis juliflora* leaf extract synthesized silver nanoparticles. Mater. Today Proc..

[24] Sheik Mydeen S, Raj Kumar R, Kottaisamy M, Vasantha VS (2020). Biosynthesis of ZnO nanoparticles through extract from *Prosopis juliflora* plant leaf: Antibacterial activities and a new approach by rust-induced photocatalysis. J. Saudi Chem. Soc..

[25] Raghavendra MP, Satish S, Raveesha KA (2009). Alkaloid extracts of *Prosopis juliflora* (Sw.) DC. (Mimosaceae) against *Alternaria alternata*. J. Biopest..

[26] Sana N, Shoaib A, Javaid A (2016). Antifungal potential of leaf extracts of leguminous trees against sclerotium rolfsiI. Afr. J. Tradit. Complement. Altern. Med..

[27] Bazie S, Ayalew A, Woldetsadik K (2014). Antifungal activity of some plant extracts against (*Colletotrichum musae*) the cause of postharvest banana anthracnose. Plant Pathol. Microbiol..

[28] Rex B, Prabhu S, Kumar JS (2019). Original article antifungal efficacies of plant extracts against *Alternaria solani* (Ellis and Martin) Jones and Grout under in vitro condition. Ann. Phytomed. Int. J..

[29] Santos E (2013). Antibacterial activity of the alkaloid-enriched extract from *Prosopis juliflora* pods and its influence on in vitro ruminal digestion. Int. J. Mol. Sci..

[30] Lima HG (2017). *Prosopis juliflora* pods alkaloid-rich fraction: In vitro anthelmintic activity on goat gastrointestinal parasites and its cytotoxicity on vero cells. Pharmacogn. Mag..

[31] Singh (2010). Antimicrobial screening of ethnobotanically important stem bark of medicinal plants. Pharmacogn. Res..

[32] Kong Q (2019). Antifungal mechanisms of α-terpineol and terpene-4-alcohol as the critical components of *Melaleuca alternifolia* oil in the inhibition of rot disease caused by *Aspergillus ochraceus* in postharvest grapes. J. Appl. Microbiol..

[33] Simionato AS (2017). The effect of phenazine-1-carboxylic acid on mycelial growth of *Botrytis cinerea* produced by *Pseudomonas aeruginosa* LV strain. Front. Microbiol..

[34] Rachitha P, Krupashree K, Jayashree GV, Gopalan N, Khanum F (2017). Growth inhibition and morphological alteration of *Fusarium sporotrichioides* by *Mentha piperita* essential oil. Pharmacogn. Res..

[35] Xing F (2014). Growth inhibition and morphological alterations of *Fusarium verticillioides* by cinnamon oil and cinnamaldehyde. Food Control.

[36] Barlian A, Anggadiredja K, Kusumorini A (2011). Damage in fungal morphology underlies the antifungal effect of lyophilisate of granular gland secretion from *Duttaphrynus melanostictus* Frog. J. Biol. Sci..

[37] Zheng J (2019). Fumigation and contact activities of 18 plant essential oils on *Villosiclava virens*, the pathogenic fungus of rice false smut. Sci. Rep..

[38] Zeidan R, Ul-Hassan Z, Al-Thani R, Balmas V, Jaoua S (2018). Application of low-fermenting yeast lachancea thermotolerans for the control of toxigenic fungi *Aspergillus parasiticus*, *Penicillium verrucosum* and *Fusarium graminearum* and their mycotoxins. Toxins (Basel).

[39] Choudhary A, Nagori BP (2013). Oral *Prosopis juliflora* treatment ameliorates inflammatory responses against carrageenan induced paw edema in rats. J. Sci. Innov. Res..

[40] Sylvester R (2015). Antibacterial activity of ethanolic extracts of *Prosopis juliflora* against gram negative bacteria. Eur. J. Exp. Biol..

[41] Dhanani T, Shah S, Gajbhiye NA, Kumar S (2017). Effect of extraction methods on yield, phytochemical constituents and antioxidant activity of *Withania somnifera*. Arab. J. Chem..

[42] Saleh I, Al-Thani R (2019). Fungal food spoilage of supermarkets' displayed fruits. Vet. World.

[43] Raja HA, Miller AN, Pearce CJ, Oberlies NH (2017). Fungal identification using molecular tools: A primer for the natural products research community. J. Nat. Prod..

[44] Eloff JN (2019). Avoiding pitfalls in determining antimicrobial activity of plant extracts and publishing the results. BMC Complement. Altern. Med..

[45] Kuete V, Karaosmanoğlu O, Sivas H, Kuete V (2017). Anticancer activities of african medicinal spices and vegetables. Medicinal Spices and Vegetables from Africa.

[46] Stefanovic OD, Tesic JD, Comic LR (2015). *Melilotus albus* and *Dorycnium herbaceum* extracts as source of phenolic compounds and their antimicrobial, antibiofilm, and antioxidant potentials. J. Food Drug Anal..

[47] Zhimo Y, Kole R, Bhutia DD, Saha J (2016). Antifungal activity of plant extracts against *Colletotrichum musae*, the post harvest anthracnose pathogen of banana cv. Martaman. Nutr. Food Sci..

